# 肺癌筛查领域指南方法学质量的系统评价

**DOI:** 10.3779/j.issn.1009-3419.2016.10.11

**Published:** 2016-10-20

**Authors:** 江 李, 凯 苏, 放 李, 威 唐, 遥 黄, 乐 王, 慧瑶 黄, 菊芳 石, 敏 代

**Affiliations:** 1 100021 北京，国家癌症中心/中国医学科学院北京协和医学院肿瘤医院，城市癌症早诊早治项目办公室 Program ofce for Cancer Screening in Urban China, National Cancer Center/Cancer Hospital, Chinese Academy of Medical Sciences and Peking Union Medical College, Beijing 100021, China; 2 100021 北京，国家癌症中心/中国医学科学院北京协和医学院肿瘤医院，胸外科 Department of Toracic Surgery, National Cancer Center/Cancer Hospital, Chinese Academy of Medical Sciences and Peking Union Medical College, Beijing 100021, China; 3 100021 北京，国家癌症中心/中国医学科学院北京协和医学院肿瘤医院，影像诊断科 Department of Diagnostic Radiology, National Cancer Center/Cancer Hospital, Chinese Academy of Medical Sciences and Peking Union Medical College, Beijing 100021, China

**Keywords:** 肺肿瘤, 筛查, 指南方法学, AGREE Ⅱ, 系统评价, Lung neoplasms, Screening, Guideline methodology, AGREE Ⅱ, Systematic review

## Abstract

**背景与目的:**

早期筛查可降低肺癌的死亡率，依据高质量的筛查指南进行实践工作是十分重要和必要的。本研究旨在了解国内外肺癌筛查指南研究的方法学质量，为我国的肺癌筛查权威指南的制定工作提供借鉴。

**方法:**

检索PubMed、Embase、Cochrane图书馆（Cochrane Library, CL）、Web of Science数据库、中国期刊全文数据库（China National Knowledge Infrastructure, CNKI）、中国生物医学文献数据库（China Bio-Medicine database, CBM）和万方等中英文共7个数据库（截至2016年2月）和相关网站有关肺癌筛查指南的所有中英文文献，按既定的纳入与排除标准，筛选文献、纳入国内外公开发布的国内外独立报告的具备方法学描述的肺癌筛查指南，采用欧洲指南研究与评估工具（Appraisal of Guidelines Research and Evaluation in Europe, AGREE Ⅱ）对质量进行评价。

**结果:**

① 共纳入11篇报告研究方法的肺癌筛查指南。②纳入指南主要由美国（81%）发布，中国和加拿大各发布1篇具备方法学描述的指南。③政府发布指南1篇，肿瘤/胸科等专业机构发布指南数9篇，未指明发布机构的指南1篇。④方法学质量评估结果：“范围和目的”领域总体评价结果平均80分，“参与人员”领域总体评价结果平均52分，“严谨性”领域总体评价结果平均50分，“清晰性”领域总体评价结果平均76分，“应用性”领域总体评价结果平均43分和“独立性”领域总体评价结果平均59分。2013年和2015年发布的指南在各领域评分较高。加拿大的指南在6个领域得分均最高。7篇指南为A推荐等级，其余4篇指南为B推荐等级。

**结论:**

① 指南发布数量呈上升趋势，主要集中在肺癌疾病负担较重的国家。②指南的制定或更新有不断打破国家和地区界限的趋势。③循证实践指南制定方法已经逐渐成为各国研究制定临床指南的趋势。④我国发布了1篇原创和独立呈现的肺癌筛查指南，质量较好。

## Abstract (en)

**Background and objective:**

Lung cancer is the most common malignancy and screening can decrease the mortality. High quality screening guideline is necessary and important for effective work. Our study is to review and evaluate the basic characteristics and methodology quality of the current global lung cancer screening guidelines so as to provide useful information for domestic study in the future.

**Methods:**

Electronic searches were done in English and Chinese databases including PubMed, the Cochrane Library, Web of Science, Embase, CNKI, CBM, Wanfang, and some cancer ofcial websites. Articles were screened according to the predefned inclusion and exclusion criteria by two researchers. Te quality of guidelines was assessed by AGREE Ⅱ.

**Results:**

At last, a total of 11 guidelines with methodology were included. Te guidelines were issued mainly by USA (81%). Canada and China developed one, respectively. As for quality, the average score in the "Scale and objective" of all guidelines was 80, the average score in the "Participants" was 52, the average score in the "rigorism" was 50, the average score in the "clarity" was 76, the average score in the "application" was 43 and the average score in the "independence" was 59. Te highest average score was found in 2013 and 2015. Canada guideline had higher quality in six domains. 7 guidelines were evaluated as A level.

**Conclusion:**

Te number of clinical guidelines showed an increasing trend. Most guidelines were issued by developed countries with heavy burden. Multi-country contribution to one guideline was another trend. Evidencebased methodology was accepted globally in the guideline development.

目前肺癌已成为人类恶性肿瘤中发病率和死亡率增长最为迅速的一种^[1]^。国际癌症研究署（International Agency for Research on Cancer, IARC）发布最新数据显示肺癌发病率已位居男性恶性肿瘤首位和女性第三位，且呈直线上升趋势；而且不论男女，肺癌死亡率均在首位^[2]^。最近研究结果指出，在人群中进行筛查能降低人群死于肺癌的概率（约降低20%）^[3]^，该结果为筛查工作人员及政府财务机构提供了较权威的筛查策略推荐指南和经济成本分析证据^[4]^。为了高效率地实现对潜在肺癌患者的早期发现、预防和治疗，以最终达到降低肺癌死亡率和整个社会与经济负担的目标，依据高质量的肺癌筛查指南进行实践工作是十分重要和必要的。

医务工作人员们对临床实践指南已不再陌生。许多国家已经制定了或者正在制定各种不同的指南，中国也正在制定自己的实践指南^[5]^。过去多数指南都是基于当地或国内各临床专家的经验和意见、教科书等制定。随着科学证据的逐渐增多，创新技术的层出不穷，患者保护意识的加强，医务人员的实践工作面临着极大的挑战。如果指南建立在严格的循证医学证据和高质量方法的基础上，采用系统的文献评价和检索方法，基于高质量的证据，并予以充分、严格和全透明的过程的推荐，在可能的情况下尽量减少偏倚产生，那么这样的指南将能够科学地成为证据转变为实践的一种策略，其临床使用则更具有可靠性^[6]^。欧洲指南研究与评估工具（Appraisal of Guidelines Research and Evaluation in Europe, AGREE）是由13个国家的研究者制定的指南研究和评价工具，主要评价指南的开发、使用和推广等，已成为世界公认评价指南质量的重要工具，目前已经更新至第二版（AGREE Ⅱ）^[7]^。

本研究调查分析了肺癌筛查指南的现状，使用AGREE Ⅱ工具评价指南方法学质量，为我国肺癌筛查指南编制工作提供借鉴，希望促进我国肺癌筛查工作在方案决策、业务操作、经济成本考虑等方面的规范化。

## 1 资料与方法

### 1.1 研究资料

计算机检索截至2016年2月的英文数据库包括PubMed、Embase、Cochrane图书馆（Cochrane Library, CL）、Web of Science数据库，中文数据库包括中国期刊全文数据库（China National Knowledge Infrastructure, CNKI）、中国生物医学文献数据库（China Bio-Medicine database, CBM）和万方数据库，同时还检索网站资源包括美国胸科医师协会数据资源（American College of Chest Physicians, ACCP）、美国国家癌症网络信息平台（National Comprehensive Cancer Network, NCCN）、美国临床肿瘤学会（American Society of Clinical Oncology, ASCO）、美国预防服务工作组（U.S. Preventive Services Task Force, USPSTF），国际肺癌研究协会（International Association for the Study of Lung Cancer, IASLC）、国际指南协作网（Guideline International Network, GIN）和中国临床指南文库（China Guideline Clearinghouse, CGC）等数据库文献资料，并手工检索已发表和通过参考文献追溯的相关指南及全文，严格依据中英文数据库主题词和关键词的检索特点制定检索策略（表 1）。

**1:** 检索策略 Search strategy

	English	Chinese
Databases	PubMed, Embase, CL, Web of Science, ACCP, NCCN, ASCO, USPSTF, IASLC, GIN	CNKI, CBM, Wanfang, CGC
Key words	Lung cancer OR "Lung Neoplasms" [Mesh] (including non-small cell lung cancer and small cell lung cancer), Screening OR "Early Detection of Cancer" [Mesh], Guideline OR recommendation	Lung Neoplasms (including non-small cell lung cancer and small cell lung cancer), Screening OR Early Detection of Cancer, Guideline OR recommendation
CL: Cochrane Library; ACCP: American College of Chest Physicians; NCCN: National Comprehensive Cancer Network; ASCO: American Society of Clinical Oncology; USPSTF: U.S. Preventive Services Task Force; IASLC: International Association for the Study of Lung Cancer; GIN: Guideline International Network; CNKI: China National Knowledge Infrastructure; CBM: China Bio-Medicine database; CGC: China Guideline Clearinghouse.		

### 1.2 纳入/排除标准

纳入公开发布的原创和更新的正常人群中肺癌筛查指南/推荐意见全文。题目中有明确“指南（guideline）”和/或“推荐意见（recommendation）”的词语且肺癌筛查指南的形式为独立报告，官方发布或者非官方发布均纳入，并且具备详细的制作流程和/或研究方法的描述。语种仅限中、英文。排除关于临床指南的介绍、评析、应用指导、应用效果评价、勘误表，排除非肺癌、非筛查类和非独立内容呈现的指南，排除其他癌种转移性质，排除翻译版本及重复收录的指南。

### 1.3 研究方法

① 指南数量统计和基本情况：将所纳入的指南根据国别（发布组织所在地区为准）、发布时间、指南名称、发布机构、是否有研究方法、更新次数与时间进行统计描述；②指南的推荐意见主要内容汇总：该部分主要汇总报告了制定方法的指南。将包括肺癌筛查时的高危人群评估指标（包括年龄、吸烟史、戒烟状态、其他疾病或遗传史等）、筛查的频率、筛查地点推荐及筛查试点的建立、筛查手段的潜在利益（包括生存率及行为改变）及潜在危害（包括过度筛查、假阳性以及筛查所引起的并发症等），以及在指南中是否鼓励受试者参与，是否有提出针对受试者的戒烟行为干预，以及制作指南过程中是否对证据等级进行分级；③指南质量初步评价：参考AGREE Ⅱ指南评价工具^[7]^，按“范围和目的、参与人员、严谨性、清晰性、应用性与独立性”6个领域23个主要条目对纳入的报告了制定方法的指南进行综合评价（表 2）。AGREE Ⅱ的每一个条目均按7分划分等级（1分代表很不同意，7分代表很同意，条目报道不能满足全部标准或条件则根据不同情况给予2分-6分）。每个领域得分等于该领域中每一个条目分数的总和，并标准化为该领域可能的最高分数。四名评价人员按照每个条目对每一篇指南进行评估判分。得出每个领域的总分，评判标准为：最大可能得分=7分（很同意）×条目数×评价者数；最小可能得分=1分（很不同意）×条目数×评价者数；领域分值是：“实际得分-最小可能得分”/“最大可能得分-最小可能得分” × 100；④指南推荐级别判定：根据6个领域的综合判断所评指南是否值得推荐应用，分为三个推荐等级：A级：积极推荐（4个及以上领域的分值≥50）、B级：推荐（3个领域的分值≥50），C级：一定条件下推荐（2个及以下领域的分值≥50）。

**2:** AGREE Ⅱ工具评价内容及分值范围 Evaluation items of AGREE Ⅱ and score range

Domains	Items	No. of items	Min score	Max score
Scope and Purpose	This deals with the potential health impact of a guideline on society and populations of patients or individuals.	3	12	84
Stakeholder Involvement	This item refers to the professionals who were involved at some stage of the development process.	4	16	112
Rigour of Development	Details of the strategy used to search for evidence should be provided including search terms used, sources consulted, and dates of the literature covered.	7	28	196
Clarity of Presentation	A recommendation should provide a concrete and precise description of which option is appropriate in which situation and in what population group, as informed by the body of evidence.	4	16	112
Applicability	There may be existing facilitators and barriers that will impact the application of guideline recommendations	3	12	84
Editorial Independence	Many guidelines are developed with external funding (e.g., government, professional associations, charity organizations, pharmaceutical companies). There should be an explicit statement that the views or interests of the funding body have not influenced the final recommendations	2	8	56

### 1.4 评价人员对评价结果的一致性判断

指南筛选采用研究人员独立判读、翻译、校对的方法，如有异议通过小组讨论解决。小组中研究人员均需具备较好的英文阅读能力和流行病学/循证医学方法的基础，并由通过组内培训对文献严格评价的方法、AGREE Ⅱ工具、评价标准及评价注意事项进行详细介绍。为保证评价结果的可靠性，随机抽取2篇纳入文献进行独立评分，共同衡量四名评价人员间对指南评价结果的一致性。

### 1.5 数据分析

文献资料管理采用Endnote软件，指标积分、统计评价采用Microsoft Excel 2010版本软件，统计评价方法采用综合评分法。

## 2 结果

### 2.1 文献检索结果

初步检索到相关文献237篇，初步符合指南标准的24篇。仔细阅读全文，按是否在指南报告中提供研究方法，最终纳入肺癌筛查领域具备研究方法的指南共11篇^[8-18]^（图 1）。

**1:**
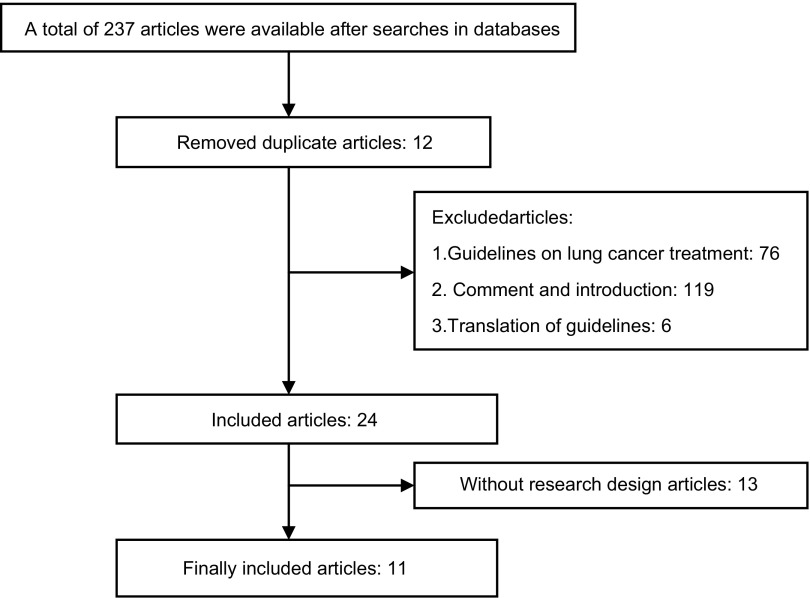
肺癌筛查指南检索流程图 Flowchart of including lung cancer screening guidelines

### 2.2 指南的基本信息

指南基本信息描述一览表见表 3，按国别、发布时间、指南名称、发布机构、是否提供研究方案、更新次数与时间分类。

**3:** 肺癌筛查指南一览表 Lists of lung cancer screening guidelines

Country	Year	Name of guideline	Publishing organization	Research design	Update times (year)
USA	2012	CT Screening recommendations^[8]^	IASLC	Yes	0
	2012	Guidance on lung cancer screening to patients and physicians^[9]^	American Lung Association (ALA)	Yes	0
	2012	Guidelines for lung cancer screening using low-dose computed tomography scans for lung cancer survivors and other high-risk groups^[10]^	American Association for Thoracic Surgery (AATS)	Yes	0
	2012	Clinical preventive service recommendation: lung cancer^[11]^	American Academy of Family Physicians (AAFP)	Yes	0
	2013	Lung cancer screening guidelines^[12]^	American Cancer Society (ACS)	Yes	0
	2013	Screening for lung cancer: diagnosis and management of lung cancer^[13]^	ACCP	Yes	0
	2013	The role of CT screening for lung cancer in clinical practice: the evidence based practice guideline^[14]^	ASCO and ACCP	Yes	0
	2014	Screening for lung cancer: U.S. Preventive Services Task Force recommendation statement^[16]^	USPSTF	Yes	0
	2014	NCCN Clinical Practice Guidelines in Oncology^[17]^	NCCN	Yes	0
Canada	2013	Screening high-risk populations for lung cancer: guideline recommendations^[15]^	Cancer Care Ontario (CCO)	Yes	0
China	2015	China national lung cancer screening guideline with low-dose computed tomography (2015 version)^[18]^	Not available	Yes	0

#### 2.2.1 指南的国别、发布时间与更新情况

2012年-2014年呈现一个具备详细方法学内容的肺癌筛查领域指南的发布高峰。发布国家主要集中在美国，占总量的81%，关注的问题包括了肺癌筛查中高危险人群筛选、方案的选择以及潜在利害等。2015年，中国发表1篇独立于肺癌诊疗规范全面版的针对肺癌筛查推荐意见。

#### 2.2.2 指南中所描述的方法

有*meta*分析/系统评价、证据分级、随机对照试验和专家意见等。2012年以来指南制定所依据的证据等级逐步提高，方法也逐渐完善。其中6篇报告了制定指南过程中所依据证据等级的分级方法。

#### 2.2.3 指南发布机构情况

指南发布机构共涉及各国肿瘤/胸科专业协会（委员会）、癌症管理中心等专业机构10篇（占91%）；未指明发布机构的指南数1篇。地区级政府层面发布1篇（9%），肿瘤/胸科等专业机构发布9篇（81%），未指明机构的1篇（9%）（表 3）。

### 2.3 指南中主要内容报告情况

11篇提供具体制作方法的指南中，均提及采用低剂量螺旋CT筛查时的高危人群选择，高危人群的年龄分为两个推荐。有8篇^[9, 11-15, 17, 18]^推荐55岁-74岁的人群接受筛查，3篇^[8, 10, 16]^推荐55岁-80岁的人群接受筛查。11篇均推荐具有30包年的吸烟史的人群接受筛查，10篇^[8, 10-18]^推荐即使现在不吸烟，但是在15年内有戒烟经历的人群接受筛查。有5篇^[8-10, 15, 17]^推荐如有其他肺部疾病史的人群接受筛查。10篇^[8-10, 12-18]^在指南中明确指出对于肺癌筛查的适当频率。7篇^[9-12, 15-17]^推荐筛查地点或者提出建立提供标准化筛查的试点。11篇均指出了筛查的潜在利益及潜在危害，具体内容需要根据具体情况对待。6篇^[9, 10, 12-14, 16]^指出需要鼓励受试者参与到筛查的项目中以体现受试者的意愿和个体化选择。6篇^[9, 10, 13, 14, 16, 17]^提出了针对高危人群的戒烟行为干预的措施。6篇^[9, 10, 13, 14, 16, 17]^对于证据进行了等级的分级（表 4）。

**4:** 肺癌筛查指南推荐意见内容一览表 List of recommendations of lung cancer screening guidelines

Guideline	High-risk population					Frequency	Settings	Potential benefits	Potential harms	Patient involvement	Smoking quitting	Evidence level
	55-74 years	55-80 years	Smoking amount 30 pack-year	Smoking quitting in 15 years	Others							
IASLC 2012^[8]^		√	√	√	√	√	-	√	-	-	-	-
ALA 2012^[9]^	√		√		√	√	√	√	√	√	√	√
AATS 2012^[10]^		√	√	√	√	√	√	√	√	√	√	√
AAFP 2012^[11]^	√		√	√	-	-	-	√	√	-	-	-
ACS 2013^[12]^	√		√	√	-	√	√	√	√	√	-	-
ACCP 2013^[13]^	√		√	√	-	√	√	√	√	√	√	√
ASCO 2013^[14]^	√		√	√	-	√	-	√	√	√	√	√
HC 2013^[15]^	√		√	√	√	√	√	√	√	-	-	-
USPSTF 2014^[16]^		√	√	√	-	√	√	√	√	√	√	√
NCCN 2014^[17]^	√		√	√	√	√	√	√	√	-	√	√
ZHOU 2015^[18]^	√		√	√	-	√	-	√	√	-	-	-
“√” refer to“mentioned”，“-”refer to“insufficient evidence”or“not mentioned”.								

### 2.4 指南质量初步评价结果

#### 2.4.1 总体质量评价结果

本研究对该11篇肺癌筛查指南进行方法学质量评价，采用AGREE Ⅱ评价工具，在“范围和目的、参与人员、严谨性、清晰性、应用性与独立性”6个领域独立计分。

“范围和目的”领域总体评价结果平均80分，“参与人员”领域总体评价结果平均52分，“严谨性”领域总体评价结果平均50分，“清晰性”领域总体评价结果平均76分，“应用性”领域总体评价结果平均43分和“独立性”领域总体评价结果平均59分。接受评价的11篇指南/推荐意见中，六个领域的平均分达到50以上者有9篇^[9, 10, 12-18]^（81%）。7篇指南^[12-18]^为A推荐等级，其余4篇指南^[8-11]^为B推荐等级（表 5）。

**5:** 肺癌筛查指南AGREE Ⅱ评估 Evaluation of lung cancer screening guideline by AGREE Ⅱ

Guideline	Domains of AGREE Ⅱ						Average score	Recommendation level
	Scope and purpose	Stakeholder involvement	Rigour of development	Clarity of presentation	Applicability	Editorial independence		
IASLC 2012^[8]^	71	41	35	55	30	55	48	B
ALA 2012^[9]^	80	54	40	65	47	22	51	B
AATS 2012^[10]^	75	37	29	85	15	58	50	B
AAFP 2012^[11]^	63	33	58	70	35	18	46	B
ACS 2013^[12]^	94	72	58	72	67	78	74	A
ACCP 2013^[13]^	78	50	47	89	61	69	66	A
ASCO 2013^[14]^	78	55	58	89	61	69	68	A
HC 2013^[15]^	90	78	78	85	42	92	78	A
USPSTF 2014^[16]^	87	50	38	78	40	86	63	A
NCCN 2014^[17]^	85	46	58	78	36	50	58	A
ZHOU 2015^[18]^	80	55	52	70	40	56	59	A
Average	80	52	50	76	43	59	60	

#### 2.4.2 不同年代指南质量评价结果

评估的11篇指南均在近5年内发布。“范围与目的”领域在2012年-2015年都达到了高于平均值的水平，“参与人员”领域在2013和2015年高于平均值水平，“严谨性”领域在2013年-2015年均高于平均值水平，“清晰性”领域在2013年和2014年高于平均值水平，“应用性”领域在2013年和2015年高于平均值水平，“独立性”领域在2013年和2014年高于平均值水平。2013年-2015年的7篇指南^[12-18]^均为A推荐等级指南；2012年的4篇指南^[8-11]^为B推荐等级指南（表 5）。

#### 2.4.3 不同国家指南质量评价结果

“范围与目的”领域中，美国发布的肺癌筛查指南的平均分为79分，加拿大的90分，中国的80分；“参与人员”领域中，美国指南的平均分为49分，加拿大的78分，中国的55分；“严谨性”领域中，美国指南的平均分为47分，加拿大的78分，中国的52分；“清晰性”领域中，美国指南的平均分为76分，加拿大的85分，中国的70分；“应用性”领域中，美国指南的平均分为44分，加拿大的42分，中国的40分；“独立性”领域中，美国指南的平均分为56分，加拿大的92分，中国的56分。A推荐等级的指南中，美国指南有5篇^[12-14, 16, 17]^，加拿大1篇^[15]^和中国1篇^[18]^（表 5）。

## 3 讨论

本研究纳入指南多数由相关癌症防控组织制定发布，具备方法学的指南发布/更新时间均在2012年之后。指南的内容、质量等会由于制定的时间、国家地区和组织不同而差异较大。临床实践指南根据制作方法主要分为基于专家共识和基于循证医学证据两个类别，2010年，世界卫生组织（World Health Organization, WHO）发布循证指南制定手册更新版后，循证临床指南更加逐渐成为指南制订的趋势和主流^[19]^。文中所纳入的具有制作方法的11篇肺癌筛查指南均是在2012年后更新和/或制作，一方面是由于2011年美国发布了具有广泛影响力的全美肺癌筛查试验（National Lung Screening Trial, NLST）的结果，使得各国肺癌筛查研究人员关注了这个严谨设计的随机对照试验所产生的高质量的证据^[20]^；另一方面则是循证指南制定手册中明确规定了指南需要依据新证据的出现，推荐在3年左右更新^[21]^。多数指南遵循制作原则，目的是产生高质量的临床推荐意见，促使更加科学有效地规范临床路径^[22]^。本研究中6篇^[9, 10, 13, 14, 16, 17]^具有证据推荐等级的循证实践指南中，涉及的内容全面，有关于肺癌筛查所考虑的问题如高危人群选择、筛查频率、筛查地点推荐及建立、筛查潜在利益和潜在危害、是否鼓励受试者参与和戒烟行为干预均有提及，而且均为A推荐等级的指南，具有较好的实用性、适用性和推广性。

制定临床指南时，还需要通过循证医学方法与证据系统研究和严格论证。本研究依据AGREE Ⅱ的6个领域对肺癌筛查指南的质量进行评价。评价的11篇指南总体质量较高，64%的指南推荐等级均为A级。在AGREE Ⅱ的6个领域中平均分数≥60的是“范围和目的”和“清晰性”，而得分较低（≤50）的领域是“应用性”，得分介于50分-60分之间的领域是“参与人员”“制定的严谨性”及“编辑的独立性”。AGREE Ⅱ评价方法不仅在指南内容的制定中强调注重临床研究的循证医学依据，同时注重指南的实用性、应用性和适用性，也就是临床实践指南如何能够实施到医疗一线工作中，促进疾病的早期诊断和早期治疗，此点在癌症防治方面尤为重要^[7]^。制定指南的参与成员由各个国家内部知名的多学科专家组成，共同撰写，针对特定临床情况的正确诊断与治疗决策，给出系统指导意见。指南如果由国家层面组织制定，则更具有科学性和权威性；如果由地方参与制定和地方机构管理实施，则更针对基层实践，具有较强的实用价值^[23]^。本研究结果显示，现有指南发布机构中区域性专门机构占35%，多由非营利官方协会发布，少数由大学或学术团体发布。其中一些肺癌筛查指南的制定或更新出现了多个国家合作的情况，这将促进指南研究的全面性、综合性、协调性和通用性。指南制定过程中往往需要政府及商业团体资助，利益冲突不可避免，进行制定组织的责任利益声明非常有必要。一方面是为了避免存在像商业团体倾向性而导致的阳性偏倚，同时也是使得所制定的指南可信度更强^[24]^。本研究评价的指南中，9篇指南在利益冲突进行了声明且评估分值大于50，可信度较高。

虽然现有的AGREE Ⅱ指南评价方法是WHO指南制定方法推荐的一种较为完善的临床指南评价模式，完全按照AGREE Ⅱ方法判别临床指南的优劣或是依据其制定指南也不能完全符合我国目前的实际情况，但是有些评价领域的指标值得我们借鉴^[25]^。本研究所评价的一篇我国推荐意见发表在英文期刊上，但是纵观国内数据库，并未有独立的肺癌筛查相关的指南，多是肺癌诊治规范等内容中包含了肺癌筛查这一个部分^[26]^，这也提示了在资源、经费、经验和时间有限的国家或地区，由于不同国家或地区间文化、组织的差异，循证指南制定方法和产出会有差异。如在指南的制定时注重指南的学术性，而忽视指南的对各类人群的推广应用以及利益声明等。通过详细阅读所评价的我国的肺癌筛查推荐意见，可以看出，我国肺癌筛查相关专家关注并遵循了循证实践指南的方法制定，充分结合了我国政府支持的肺癌筛查地域性项目所获得的数据，使得推荐意见具有独立性和地域的指导性。这也提示，借鉴国外高质量指南的制订标准、规范证据与当地疾病负担、遗传特点及卫生资源条件相结合，采用循证理念制定出高质量证据支持的本土化指南是我国标准版癌症筛查指南的发展方向。

关于评价工具，本研究是首次使用AGREE Ⅱ评价肺癌筛查指南/推荐意见。由于研究人员对英文版的AGREE Ⅱ工具的理解差异，虽然在项目前期进行了统一的培训而且预评价的一致性较好，但对AGREE Ⅱ工具中条目评分体系仍然存在异议。我们将会在今后的研究中结合不同的指南评价工具进行评价，尽量避免偏倚发生。AGREE Ⅱ在国际上已经具有较高权威性，但其只是评价指南研究方法学质量，对具体实践的内容尚未有评价标准，且我国目前癌症筛查指南能够依据的本土化高质量临床试验证据较少，与国际化标准仍有一定差距，故所纳入的特别是结合我国国情制订的循证实践指南较少。除此之外，本研究局限性在于检索文献过程中限定了语种（中文和英文），因不同的国家地区会有针对性的肺癌筛查指南的发布，语种的限制可能会造成不完全的检索。我们会在今后的研究中，尽量纳入其他语种的肺癌筛查领域指南，通过专业人员进行翻译从而提供全球化多语种的指南方法学质量评价。

目前提供研究方案的肺癌筛查指南的总体质量较高，且集中在近3年发布。肺癌筛查指南在“应用性”领域需要加强。建议由权威机构筹划，制定出重点突出的、符合我国国情的癌症筛查循证实践指南完整版并定期更新，适应医疗技术的发展及临床的规范化，从而有力地促进我国癌症早期诊断和早期治疗项目的工作效率与成果。
